# α-Actinin-1 in Megakaryocytes: Its Structure, Interacting Proteins and Implications for Thrombopoiesis

**DOI:** 10.3390/biomedicines13102479

**Published:** 2025-10-11

**Authors:** Lanlan Wu, Zhiqun Song, Yulan Zhou, Jiansong Huang, Xiaoxia Huang

**Affiliations:** 1 Department of Nursing, Department of Emergency Medicine, The Second Affiliated Hospital, Zhejiang University School of Medicine, Hangzhou 310003, China; 2 Department of Blood Transfusion, Jiangsu Province People’s Hospital and the First Affiliated Hospital of Nanjing Medical University, Nanjing 210029, China; 3 Department of Hematology, The First Affiliated Hospital of Nanchang University, Nanchang 330006, China; 4 Key Laboratory of Hematologic Malignancies, Diagnosis and Treatment, Department of Hematology, The First Affiliated Hospital, Zhejiang University School of Medicine, Hangzhou 310003, China

**Keywords:** thrombopoiesis, megakaryocyte, platelet, α-actinin-1

## Abstract

Mutations in the *ACTN1* gene, which encodes the cytoskeletal protein α-actinin-1, have been implicated in the etiology of autosomal dominant congenital macrothrombocytopenia. α-Actinin-1 is a member of the spectrin superfamily and is essential for key physiological processes in megakaryocytes and platelets. The pathophysiological mechanisms by which α-actinin-1 mutations lead to macrothrombocytopenia have been attributed to alterations in actin organization, increased binding affinity of α-actinin-1 to actin filaments, and modulation of integrin αIIbβ3 signaling. In previous studies, we utilized megakaryocyte-specific α-actinin-1 knockout (PF4-*ACTN1*^−/−^) mice to explore the influence of α-actinin-1 on megakaryocyte and platelet function. Despite these efforts, the precise mechanisms remain inadequately understood. To advance our understanding and clarify the role of α-actinin-1 in thrombopoiesis, we first delineated the functions of α-actinin-1 in megakaryocytes and platelets, followed by a comprehensive overview of the proteins known to interact with α-actinin-1. As a pivotal scaffold protein, α-actinin-1 interacts with a complex network of partners, including integrin αIIbβ3, and actin filaments, to modulate cytoskeletal dynamics, megakaryocyte maturation, and proplatelet formation. In addition to its well-documented proteins that interact with α-actinin-1 within megakaryocytes and platelets, α-actinin-1 also associates with proteins outside the megakaryocytic lineage, such as cytohesin-2 and MOB1, which have been predominantly examined in other cellular contexts. These varied interactions imply that α-actinin-1 may influence megakaryocyte and platelet functions through multiple mechanisms. This review provides a comprehensive synthesis of current knowledge regarding the structure, binding partners of α-actinin-1, and essential roles of α-actinin-1 in thrombopoiesis.

## 1. Introduction

Inherited thrombocytopenia is a highly heterogeneous disorder primarily attributed to genetic abnormalities affecting megakaryocytes (MKs) and platelets [1,2]. Clinically, this condition is characterized by a decreased platelet count and compromised platelet function, which may result in impaired hemostasis [1]. To date, mutations responsible for inherited thrombocytopenia have been identified in more than 30 genes. These genes are categorized into six groups on the basis of their subcellular localization and function: (1) genes related to cytoskeletal components [3,4,5,6,7,8,9,10]; (2) genes associated with glycoprotein adhesion receptors [11,12,13,14]; (3) genes related to transcription factors [15,16,17,18,19,20,21]; (4) genes involved in platelet granules [22,23]; (5) genes related to signal transduction [24,25,26,27,28]; and (6) other genes, including *ABCG5/ABCG8*, *RBM8A*, and *ANKRD26* [29,30,31]. Inherited thrombocytopenia is categorized into three subgroups on the basis of the mean platelet volume: large, normal, or small. Among these, congenital macrothrombocytopenia (CMTP) is particularly significant and is characterized by the presence of large platelets and reduced platelet counts. Recent studies have identified abnormalities in the *ACTN1* gene as a contributing factor to CMTP [32,33,34,35,36,37,38,39]. Patients with these genetic aberrations typically exhibit decreased platelet counts, increased platelet volumes, and minor impairments in certain platelet functions, such as a 37% reduction in adenosine diphosphate (ADP)-induced aggregation, whereas collagen- and ristocetin-induced aggregation remains unaffected. The clinical manifestations range from asymptomatic to severe bleeding [36]. In a study of 13 Japanese CMTP pedigrees, *ACTN1* gene mutations were identified in six pedigrees (46%) [32]. These mutations represent the fourth most common cause of CMTP in the Japanese population, accounting for 5.5% of cases [32]. The *ACTN1* gene encodes the protein α-actinin-1. This review provides a comprehensive overview of the current knowledge regarding α-actinin-1 in MKs, including its fundamental structure, associated binding proteins, and pivotal role in thrombopoiesis. The term ‘α-actinin-1’ refers specifically to this isoform, whereas the general term ‘α-actinin’ is used when unspecified isoforms from earlier research are cited.

## 2. Thrombopoiesis

**Figure 1:**
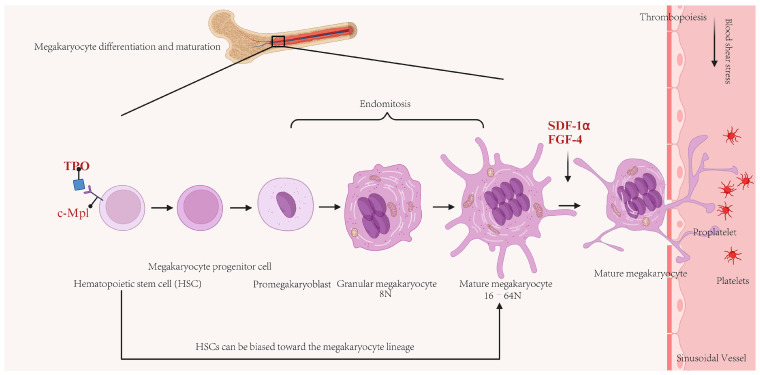
Schematic of the process of megakaryocyte (MK) maturation, polyploidization, and platelet production. In the bone marrow (BM), classical model, hematopoietic stem cells are induced by thrombopoietin (TPO)/c-Mpl signaling and undergo multiple rounds of endomitosis to achieve polyploidization. Concurrently, mitochondria provide energy for rapid cytoplasmic expansion, ultimately leading to differentiation into mature MKs. Under the coordinated action of cytokines such as stromal cell-derived factor-1α (SDF-1α) and fibroblast growth factor-4 (FGF-4), mature MKs migrate to sinusoidal capillaries rich in blood vessels. An alternative model suggesting the presence of MK/platelet-biased HSCs within the HSC population. Under the influence of blood flow shear stress, proplatelets extending from mature MKs are released into the circulating blood, where they become functional platelets.

Platelets play important roles in physiological and pathological processes, such as hemostasis, thrombosis, inflammation, and tumor metastasis [40]. These enucleated cell fragments are derived from MKs, with each MK capable of producing approximately 1000 to 3000 platelets. The production of platelets can be conceptually divided into two distinct stages. The initial stage involves the maturation of MKs. According to the classical model of megakaryopoiesis, hematopoietic stem cells (HSCs), which are located in the osteoblastic niche—a proliferative microenvironment within the bone marrow (BM)—differentiate into MK-erythroid progenitor cells, which subsequently evolve into distinct MK progenitor cells [41]. Under regulation by chemokines, growth factors (primarily thrombopoietin), stromal cells, and other regulatory factors, MK progenitor cells differentiate into mature MKs (stage III MKs) through two intermediary stages: megakaryoblasts (stage I MKs) and promegakaryocytes (stage II MKs), thereby preparing for platelet production [42]. Furthermore, recent studies have proposed an alternative model suggesting the presence of MK/platelet-biased HSCs within the HSC population [43,44,45]. During the process of MK maturation, these cells undergo significant enlargement accompanied by extensive nuclear proliferation and the biosynthesis of platelet-specific granules. Additionally, MKs expand their cytoplasmic contents and develop a demarcation membrane system (DMS) to facilitate the formation of the plasma membranes necessary for platelet production [46]. The subsequent stage encompasses proplatelet formation and platelet release, which occur within several hours. Under regulation by stromal cell-derived factor 1-α (SDF-1α) signaling, mature MKs (ranging from 8 to 64 N ploidy) migrate from the BM niche to the capillary-rich vascular niche and the lung microcirculation, where they extend their cytoplasm to form branched structures known as proplatelets [47,48]. Under conditions of shear and turbulent flow, platelets are subsequently released from these proplatelets into the circulation [49]. Figure 1 shows a schematic of the process of MK maturation, polyploidization, and platelet production.

The microtubule system underpins the extensions of proplatelets and the discoid shape of platelets. In contrast, the actin filament system in nonactivated platelets remains inactive. However, platelet activation triggers a dynamic reorganization of the actin filament system, which facilitates morphological changes in response to external stimuli. Cytoskeletal reorganization plays a crucial role not only in the induction of proplatelet formation and platelet release from MKs but also in ensuring the proper functionality of platelets within the circulatory system. Cytoskeleton rearrangement facilitates the redistribution of organelles and granular components within the MK cytoplasm and promotes the extension of the cytoplasm to form proplatelets, which ultimately release platelets. The functions of platelets, including spreading, aggregation, clot retraction, and the release of granular contents, are also dependent on cytoskeletal rearrangement. Cytoskeletal proteins determine the structures of MKs and platelets, as well as their capacity to respond to various stimuli. Accumulating evidence suggests that the actin filament-based cytoskeleton [50] and certain actin-binding proteins, such as twinfilin 2a [51], cofilin 1 [52], filamin A [53], L-plastin [41], dematin [54] and nonmuscle myosin IIA [55], and controlling cytoskeletal dynamic proteins, such as Rho GTPases [56,57], play critical pathophysiologic roles in megakaryopoiesis, platelet biogenesis, and function. Mutations or deletions in genes encoding cytoskeletal-related proteins can lead to conditions such as CMTP [58]. Specifically, mutations in the *MYH9* gene, which encodes nonmuscle myosin heavy chain IIA, result in macrothrombocytopenia [58,59]. Mutations in the *PLNA* gene are associated with reduced platelet counts, increased platelet volumes, and enlarged α granules [60]. Furthermore, genes such as *TUBB1* [61], *WASP* [62], *DIAPH1* [63], *TRPM7* [10], *TMP4* [64], and *ACTN1* [35] influence platelet production and function through reorganization of the cytoskeleton. Specifically, mutations in the actin-binding domain (ABD), rod domain, and calmodulin-like domain (CaMD) of α-actinin-1 result in the loss of normal function, leading to CMTP [32,33,34,35,36,38,39]. Motoko et al. recently reported that an SLR2 mutation (L395Q) in α-actinin-1 is responsible for CMTP [36].

## 3. The Role of α-Actinin-1 in MKs and Platelets

The understanding of α-actinin-1 functions within MKs has undergone significant evolution over time. Initial investigations focused predominantly on the general role of cytoskeletal proteins in MKs. However, advancements in molecular biology techniques have facilitated more detailed examinations of the specific functions of α-actinin-1 in MK biology. In 2007, Raslova et al. employed a gene profiling approach to demonstrate that the expression of the *ACTN1* gene is upregulated during polyploidization and MK differentiation [65]. Furthermore, α-actinin has been identified as essential for the regulation of cytokinesis in both yeast and mammalian cells [66]. In 1978, Fujiwara et al. first demonstrated that α-actinin localizes to the cleavage furrow alongside myosin during cytokinesis in mammalian cells, suggesting that it plays a role in anchoring the contractile ring to the plasma membrane [67]. In 2013, Elagib et al. revealed that the knockdown of α-actinin-1 resulted in reduced megakaryocytic polyploidization [68]. The expression of α-actinin-1 is upregulated during MK differentiation through the activation of calpain 2, which in turn activates the positive transcription elongation factor b (p-TEFb) pathway, underscoring its critical role in terminal megakaryopoiesis and platelet biogenesis [68]. In the same year, a pivotal historical discovery was made with the identification of mutations in α-actinin-1 among patients with CMTP [32]. This discovery was significant, as it established a direct association between α-actinin-1 and disorders related to MKs. Among the MKs, α-actinin-1 plays a critical role in the crosslinking of actin filaments, a process essential for maintaining proper actin filament-based cytoskeleton structure and facilitating dynamic remodeling during proplatelet formation. Proplatelet formation is the mechanism by which MKs extend cytoplasmic projections that subsequently fragment into platelets. Mutations in the *ACTN1* gene associated with CMTP disrupt normal actin bundling, resulting in a disorganized actin filament-based cytoskeleton. This structural defect leads to abnormal proplatelet morphology, characterized by fewer but larger proplatelet tips, which correlates with the production of fewer and larger platelets, a condition known as macrothrombocytopenia, in affected individuals. Therefore, α-actinin-1 is indispensable for efficient platelet production and the maintenance of actin filament-based cytoskeleton integrity in MKs.

α-Actinin-1 plays critical roles in both MKs and platelets, primarily through its function as an actin crosslinking protein that regulates cytoskeletal dynamics. In MKs, α-actinin-1 is involved in endomitosis, a process essential for polyploidization and maturation [68,69]. It contributes to the formation of the contractile ring during cytokinesis, and its dysregulation can lead to defective polyploidization and impaired MK enlargement. Additionally, α-actinin-1 is implicated in proplatelet formation, where it helps regulate the branching and extension of proplatelets. Mutations in α-actinin-1 result in reduced proplatelet tip numbers and increased tip size, ultimately leading to macrothrombocytopenia [32,39].

In platelets, α-actinin-1 is crucial for activation, adhesion, and shape changes. It facilitates actin filament bundling in pseudopods and filopodia during platelet activation, and its phosphorylation status modulates interactions with integrins such as αIIbβ3, influencing platelet aggregation and the response to shear stress. α-Actinin-1 also anchors signaling complexes with GPIb-IX-V, contributing to platelet adhesion under flow conditions [70]. Furthermore, it localizes to actin nodules and podosome-like structures, enhancing platelet aggregate stability [71]. Its ability to sense mechanical forces and regulate cytoskeletal reorganization underscores its importance in both platelet production and function.

Our study utilized MK-specific α-actinin-1 knockout (platelet factor 4 (PF4)-*ACTN1*^−/−^) mice and demonstrated that these mice presented with reduced platelet counts, compromised platelet functionality, diminished thrombus formation, and impaired mitochondrial activity [72]. In these mice, there is a reduction in the number of MKs within the BM, accompanied by abnormalities in MK ploidy, characterized by an increased proportion of 2–4 N MKs and a decreased proportion of 8–32 N MKs [72]. Furthermore, PF4-*ACTN1*^−/−^ platelets exhibit reduced spreading, clot retraction, aggregation, integrin αIIbβ3 activation, and P-selectin exposure in response to various agonists [72].

## 4. The Structure and Expression of α-Actinin-1

**Figure 2:**
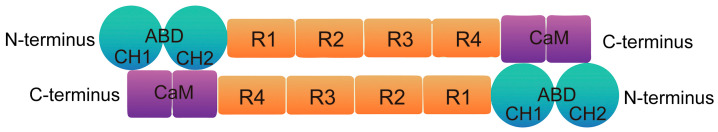
Schematic diagram of the α-actinin-1 dimer. The α-actinin-1 monomer consists of three conserved domains: the N-terminal actin-binding domain (ABD), which is composed of two CH (calponin homology) subdomains; the central rod domain, which is made up of four spectrin-like repeat (R) subdomains; and the C-terminal calmodulin (CaM) domain, which contains two Ca^2+^-binding E-helix and F-helix (EF)-hand subdomains. Two identical monomers assemble in an antiparallel manner to form an α-actinin-1 dimer.

α-Actinin-1 is a cytoskeletal protein belonging to the spectrin superfamily of actin-binding proteins [32,73]. Among the four identified α-actinin isoforms, α-actinin-1 is categorized as a nonmuscle isoform because of its extensive expression in nonmuscle cells and its sensitivity to calcium. Structurally, α-actinin-1 has a conserved domain architecture characteristic of all α-actinin isoforms: it possesses an N-terminal actin-binding domain (ABD) comprising two calponin homology (CH) domains (CH1 and CH2), a connecting segment (neck), a central rod domain composed of four spectrin repeats (SR1–SR4) that facilitate antiparallel dimerization, and a C-terminal calmodulin-like (CaM) domain containing E-helix and F-helix (EF)-hand motifs (Figure 2). The protein functions as an antiparallel homodimer, enabling it to crosslink actin filaments at both ends, thereby contributing to the organization and stability of the actin cytoskeleton. The actin-binding domain (ABD) region encompasses at least three primary actin-binding sites (ABSs) and facilitates the cross-linking of actin filaments at a 1:10 ratio within bundles [74,75]. Additionally, the ABD region can engage in direct or indirect interactions with integrins. Specifically, the ABD of α-actinin-1 is crucial for F-actin binding, with significant actin-binding sites located within the CH1 domain and the linker region between CH1 and CH2. The central rod domain plays a role in dimerization and imparts structural stability, whereas the CaM domain modulates actin-binding activity in a calcium-dependent fashion. The CaMD region comprises four EF-hand motifs (EF1/2 and EF3/4) [32,76], which have the capacity to bind calcium ions. In nonmuscle isoforms, such as α-actinin-1, the binding of calcium induces conformational changes that influence the efficiency of actin crosslinking.

In mammals, the α-actinin family comprises four members: α-actinin-1 (MIM 102575, human chromosome 14q24.1), α-actinin-2 (MIM 102573, human chromosome 1q43), α-actinin-3 (MIM 102574, human chromosome 11q13.2), and α-actinin-4 (MIM 604638, human chromosome 19q13.2) [77]. These α-actinins are categorized into two distinct classes on the basis of their sensitivity to calcium ions (Ca^2+^): Ca^2+^-sensitive and Ca^2+^-insensitive [77]. Specifically, α-actinin-1 and α-actinin-4 exhibit Ca^2+^ sensitivity and are broadly expressed in nonmuscle cells, whereas α-actinin-2 and α-actinin-3 are Ca^2+^-insensitive and predominantly localized to muscle cells [77]. α-Actinin-1 is ubiquitously expressed across various tissues, including platelets, endothelial cells, fibroblasts, and epithelial cells. It is localized to stress fibers, focal adhesions, and adherens junctions, where it functions as a crucial scaffold linking the actin filament-based cytoskeleton to transmembrane receptors such as integrins and cell adhesion molecules. In platelets, α-actinin-1 is particularly abundant and plays vital roles in platelet activation, aggregation, and MK maturation. Its expression and subcellular localization are dynamically regulated during cellular processes such as migration, adhesion, and cytokinesis.

Mutations in the *ACTN1* gene frequently result in either an increased affinity for actin binding or a disruption of structural integrity, culminating in actin filament-based cytoskeleton disorganization and compromised platelet functionality. Furthermore, the expression of α-actinin-1 is subject to modulation in various cancers, although its precise role in oncogenesis is not as well understood as that of α-actinin-4 [73]. Overall, α-actinin-1 is a multifunctional protein that plays a critical role in actin filament-based cytoskeleton dynamics, mechanical stability, and cellular signaling, with its expression and function being meticulously regulated across diverse cell types and physiological contexts. Several comprehensive reviews have provided an in-depth examination of the structure and functions of α-actinins [73,75,78,79,80]. The binding partners that can interact with α-actinin-1 are detailed in Table 1 and Table 2.

## 5. Proteins That Interact with α-Actinin-1

### 5.1. Integrin αIIbβ3 (Also Known as Glycoprotein GPIIb/IIIa)

Integrin αIIbβ3, comprising the integrin αIIb and β3 subunits, serves as the principal membrane receptor in MKs and platelets, facilitating rapid and bidirectional signal transduction across the plasma membrane [40,103]. In its resting state, integrin αIIbβ3 adopts an inactive conformation. Upon stimulation of platelets by various agonists, including thrombin, ADP, thromboxane A2 (TXA2), and collagen, integrin αIIbβ3 undergoes inside-out signaling, also referred to as integrin αIIbβ3 activation. The binding of activated integrin αIIbβ3 to its ligand initiates outside-in signaling and promotes integrin αIIbβ3 clustering, ultimately triggering a cascade of intracellular signaling events. These events include the phosphorylation of Y747, T753, and Y759 in the cytoplasmic tail of integrin β3; the activation of Src family kinases (SFKs) and spleen tyrosine kinase (Syk); and the subsequent activation of downstream signaling pathways associated with SFKs and Syk [40,104]. Current models suggest that Syk interacts with immunoreceptor tyrosine-based activation motif (ITAM)-containing receptors that interact, in turn, with the integrin [105]. Integrin αIIbβ3 outside-in signaling is also important for thrombopoiesis, including MK maturation and proplatelet formation [46,106]. The structure, signaling, and function of integrin αIIbβ3 have been summarized in several comprehensive reviews [40,104,107,108,109,110], which can be referenced further.

The bidirectional signal transduction of integrin αIIbβ3 is thought to be mediated by direct or indirect interactions of integrin αIIbβ3 cytoplasmic tails with intracellular signaling proteins. The cytoplasmic tail of human integrin β3 is longer than the cytoplasmic tail of human integrin αIIb and contains three key amino acid residues, namely, tyrosine Y747, threonine T753, and tyrosine Y759, which can be phosphorylated [111,112,113]. Considerable progress in understanding the proteins that interact with integrin αIIbβ3 cytoplasmic tails and regulate the bidirectional signaling of integrin αIIbβ3 has been achieved in recent years. For example, vacuolar protein sorting-associated protein 33B (VPS33B) [114], talin rod [115], Src [116,117], and Gα13 [118,119] are involved in integrin αIIbβ3 outside-in signaling, and integrin-linked kinase (ILK) [120], β3-endonexin [121], talin head [122], and kindlin-3 [123] participate in integrin αIIbβ3 inside-out signaling. Notably, phosphorylation of T753, Y747, and Y759 in the integrin β3 cytoplasmic tail can affect interactions between the integrin β3 cytoplasmic tail and intracellular signaling proteins, which further regulate integrin αIIbβ3 bidirectional signal transduction. Phosphorylation of T753 on the TST753 motif of the integrin β3 cytoplasmic tail can interfere with the binding of the Shc protein and SH2-containing proteins to the integrin β3 cytoplasmic tail [113]. Thrombin stimulation can cause Y747 and Y759 phosphorylation in the integrin β3 cytoplasmic tail in platelets. Some questions remain regarding the molecular mechanism by which α-actinin-1 regulates integrin αIIbβ3 bidirectional signal transduction; for example, does tyrosine phosphorylation of the integrin β3 cytoplasmic tail affect the interaction between the integrin β3 cytoplasmic tail and α-actinin-1 in integrin αIIbβ3 bidirectional signal transduction?

α-Actinin-1 is involved in the bidirectional signal transduction of integrin αIIbβ3 through its interactions with the integrin β3 cytoplasmic tail. Specifically, α-actinin-1 can bind to the F727AK FEE ERA R736 sequence within the human integrin β3 cytoplasmic tail, contributing to the formation of focal adhesions and facilitating the linkage between integrins and the cytoskeleton [124,125]. At cell adhesion sites, such as focal adhesions, α-actinin-1 serves as a linker between F-actin and integrins. The ABD of α-actinin-1 can bind to integrins either directly or with the assistance of associated proteins such as talin, vinculin, zyxin, and tensin [85,91]. Consequently, α-actinin-1 is implicated in integrin αIIbβ3-mediated bidirectional signal transduction in platelets [75,126]. Under shear stress conditions, α-actinin dissociates from the β3 integrin tail [127]. Nonetheless, the precise role of α-actinin-1 in integrin αIIbβ3 bidirectional signaling remains inadequately characterized. Investigations utilizing a Chinese hamster ovary (CHO) cell model have demonstrated that the interaction between α-actinin-1 and the integrin β3 cytoplasmic tail contributes to maintaining integrin αIIbβ3 in an inactive state [124,125]. Recent research has indicated that α-actinin acts as a negative regulator, sustaining integrin αIIbβ3 in a low-affinity state [126]. Through coimmunoprecipitation assays, Tadokoro et al. reported that α-actinin is constitutively associated with integrin αIIbβ3 in resting platelets [126]. Upon stimulation of platelets with protease-activated receptor 1-activating peptide (PAR1-AP), α-actinin dissociates from integrin αIIbβ3. Remarkably, α-actinin reassociated with integrin αIIbβ3 20 min after PAR1-AP stimulation. Potential α-actinin binding sites on the human integrin β3 cytoplasmic tail partially overlap with talin binding sites. When associated with integrin αIIbβ3, α-actinin may obstruct talin access to the integrin β3 cytoplasmic tail [124,126]. α-Actinin and talin compete for binding to the integrin β3 cytoplasmic tail [128]. Recent research has indicated that the activation of integrin αIIbβ3 leads to an increase in cytoplasmic Ca^2+^ levels in platelets, subsequently activating calpain [129]. Activated calpain cleaves talin into head and rod domains [129]. Integrin-binding site 2 (IBS2) within the talin rod can interact with the E726FA KFE EE733 sequence of the human integrin β3 cytoplasmic tail, contributing to the formation of focal adhesions and facilitating the linkage between integrins and the cytoskeleton [130]. A study conducted by Roca-Cusachs et al. using mouse embryonic fibroblasts (which express integrin αvβ3 but not integrin αIIbβ3) demonstrated that α-actinin-1 and talin compete for binding to the integrin β3 cytoplasmic tail during focal adhesion maturation [82]. Considering the structural and functional disparities between integrins αvβ3 and αIIbβ3, the potential competitive interaction between α-actinin-1 and talin rods in the bidirectional signal transduction of integrin αIIbβ3 remains unresolved. Shams et al. demonstrated that α-actinin disrupts integrin αIIbβ3 signaling by inducing a kink in the transmembrane domain of integrin β3 [128]. In the human megakaryoblast cell line CMK, where α-actinin-1 expression was reduced via short hairpin RNA (shRNA) technology, decreased α-actinin levels increased integrin αIIbβ3 activation in response to PAR1-AP [126]. Conversely, the overexpression of wild-type α-actinin inhibited PAR1-AP-induced integrin αIIbβ3 activation [126].

In MKs expressing the human α-actinin-1 Q32K or V105I mutants, the number of proplatelets per MK decreased, although the size of the proplatelets increased [32]. Integrin αIIbβ3 and its bidirectional signaling are crucial for platelet production [46,131]. Mutations leading to constitutively activated integrin αIIbβ3, such as the R995W mutation in the integrin αIIb subunit or the D723H mutation in the integrin β3 subunit, have been associated with thrombocytopenia [132,133]. The activation of integrin αIIbβ3 by IgM autoantibodies leads to a decrease in platelet production [134]. The absence of Rasa3 or filamin A has been shown to result in the upregulation of integrin αIIbβ3 signaling, consequently diminishing platelet production [135,136]. Conversely, the inhibition of several key kinases within the integrin αIIbβ3 signal transduction pathway, specifically through the use of SU6656 (a Src inhibitor) and Y27632 (a Rho-related kinase inhibitor), has been shown to increase proplatelet production [135,137]. Our findings indicate that the inhibition of integrin αIIbβ3 outside-in of the signal transduction pathway using ibrutinib facilitates proplatelet production [46]. Collectively, these studies suggest that α-actinin-1 may play a regulatory role in integrin αIIbβ3 signaling, thereby influencing platelet production.

### 5.2. Actin

The current understanding of the function or role of α-actinin-1 in the actin filament-based cytoskeleton is not derived from MKs or platelets but rather from various cells, including CHO cells, fibroblasts and HeLa cells, which indicates that α-actinin-1 cross-links actin into bundles. CHO cells were transfected with different α-actinin-1 mutants (Q32K, R46Q, V105I, R197W, E225K, R738W, R752Q, or L395Q mutant) [32,36]; COS-7 cells were transfected with an R46Q mutant [34]; fibroblasts were transfected with individual D22N, R46W, G251R, D666V, T737N, G764S, or E769K mutants [35]; and HeLa cells were transfected with individual Q32K, R46Q, V105I, E225K, R738W, or R752Q mutants [76]. All of these findings showed that the α-actinin-1 mutants caused varying degrees of disorganization of the actin filaments in the mutant-transduced cells. The α-actinin-1 mutants colocalized with less fine, shortened actin filaments. The unbound α-actinin-1 mutants were coarsely distributed within the cytoplasm, and actin–filament organization was deregulated. Consistently, in vitro assays further confirmed that these mutants display increased binding affinity for F-actin and altered actin-binding properties, which aligns with the cytoskeletal disorganization observed in mutant-transfected cells [32,36,76]. However, the aforementioned studies have two key limitations: (1) none of the cell lines used were MK-derived, precluding direct extrapolation to MK or platelet biology, and (2) all tested cells retained endogenous wild-type α-actinin-1 expression, which may mask the true phenotypic effects of the mutants due to potential functional compensation.

In addition to its interaction with actin, α-actinin has been described as a major platform involved in interactions with many cytoskeletal proteins and signaling regulatory proteins. At the site of cell–cell and cell–matrix adhesions, α-actinin interacts with cell surface receptors such as integrin receptors and the *N*-methyl-D-aspartate (NMDA) receptor. In focal adhesions, α-actinin also binds to zyxin, capZ and vinculin to induce mechanochemical conversion. In muscle, α-actinin interacts with titin and Z-disk proteins, such as z-band alternatingly structured protein (ZASP) and myotilin. In addition, several intracellular molecules, such as the protein kinases PKN and mitogen-activated protein kinase kinase kinase 1 (MEKK1); PDZ domain proteins, such as ZASP/cypher; and zinc-finger proteins, such as ALP, interact with α-actinin, and these interactions contribute to the most important contractile functions in muscle cells [138]. Zyxin, a focal adhesion protein widely expressed in eukaryotes that regulates actin filament-based cytoskeleton remodeling, plays important roles in MK and platelet biology. Zyxin-deficient (Zyx^−/−^) mice present increased numbers of immature Stage I MKs and reduced numbers of mature Stage II/III MKs [139].

### 5.3. C-Terminal LIM Domain Protein of 36 kDa (CLP36)

Bauer et al. reported that human CLP36, a PDZ-domain and LIM-domain protein, binds to spectrin-like repeats 2 and 3 within the rod domain of α-actinin-1 and associates with actin filaments and stress fibers in activated platelets and endothelial cells [86]. CLP36 binds to α-actinin-1 in resting platelets, and the CLP36/α-actinin-1 complex is translocated to the newly formed actin filament-based cytoskeleton in activated platelets [86]. This study suggested that by binding to α-actinin-1, CLP36 may direct it to specific actin structures (such as stress fibers) and potentially modulate its function, such as enhancing its actin cross-linking and bundling activities, thereby playing a role in cytoskeletal remodeling during changes in cell shape and migration. The CLP36-α-actinin-1 complex represents a novel functional unit that regulates actin filament-based cytoskeleton dynamics and is likely to contribute to the final crucial steps of platelet generation by MKs.

### 5.4. Integrins β2 and β1 and Integrin α5

Several integrins, including LFA-1 (integrin αLβ2) and Mac-1 (integrin αMβ2), share a common β2 subunit and are present exclusively on leukocytes [89]. Circulating leukocytes are nonadherent but bind tightly to endothelial cells following the activation of leukocyte integrins. The increased avidity of leukocyte integrins for endothelial ligands is regulated, in part, by interaction of the β2 subunit cytoplasmic tail with the actin filament-based cytoskeleton. The integrin αLβ2 is linked to the actin filament-based cytoskeleton through α-actinin-1 [90]. The association of α-actinin with integrins may stabilize the actin filament-based cytoskeleton and promote firm leukocyte adhesion to and migration across the endothelium [140]. The activation of neutrophils results in an α-actinin-mediated association between integrin β2 and actin filaments [141]. α-Actinin binds to the cytoplasmic domain of integrin αLβ2 with an extended low-intermediate affinity conformation of the large extracellular domain [142]. The affinity of α-actinin for the integrin β2 tail is regulated by a conformational change in the β2 tail, which exposes a cryptic α-actinin binding domain. A positive domain and an inhibitory domain within the β2 tail regulate α-actinin binding. Residues 736–746 of the β2 tail are necessary and sufficient for α-actinin binding; however, residues 748–762 of the β2 tail inhibit the constitutive association of the β2 tail with α-actinin [140]. In contrast, α-actinin is constitutively associated with β1 integrins in fibroblasts [81]. The binding of α-Actinin to the full-length integrin β1 tail was 9.7-fold greater than that to the full-length integrin β2 tail [140]. It has been reported that α-actinin can cooperate with talin to activate integrin α5β1 by restricting cytoplasmic tail movement [128]. Integrin α5 is a member of the integrin protein family. Wang et al. reported that α-actinin-1 interacts with integrin α5 to promote cell proliferation, invasion, and epithelial–mesenchymal transformation in head and neck squamous cell carcinoma [83].

Integrin β2 is not expressed on MKs or platelets. Integrin β1 plays multifaceted roles in MK and platelet biology through its association with various α subunits, tightly regulating core processes, including adhesion, niche migration, maturation, and platelet production. Integrin α5β1 is pivotal for MK adhesion to fibronectin (FN), a key extracellular matrix (ECM) component in the BM microenvironment. This Integrin α5β1-FN interaction directly supports MK expansion and maturation. Ex vivo studies have shown enhanced MK growth when cultured on FN, whereas antibody-mediated inhibition of the α5 subunit abrogates this promegakaryopoietic effect [143]. Pathologically, in primary myelofibrosis (PMF) driven by the JAK2V617F mutation, MKs exhibit upregulated α5β1 expression and activation, leading to abnormally increased adhesion to FNs and uncontrolled MK proliferation; crucially, targeting the α5 subunit with neutralizing antibodies reduces the number of MKs both in vitro and in vivo, highlighting the therapeutic potential of disrupting this β1-dependent axis [144]. In addition to FN adhesion, integrin α5β1 governs MK localization across BM niches: in immature MKs, activated integrin α5β1 mediates retention in the endosteal niche to initiate early maturation [145]. Integrin β1 also modulates how MKs respond to collagen through its association with the α2 subunit to form Integrin α2β1. Specifically, when α2β1 is constitutively activated in MKs, it leads to a ligand-dependent reduction in the amount of α2β1 present on the MK surface. This decrease in surface α2β1 is important because it stops MKs from sticking too early (prematurely) to the collagen-rich environment of the BM. By avoiding this premature adhesion, MKs can move smoothly and unobstructed to the vascular niche—the site where they complete the process of thrombopoiesis (platelet production) properly [146]. Conditional knockout of integrin α2β1 in murine MKs results in a significant reduction in the mean platelet volume (MPV), demonstrating the role of integrin β1 in fine-tuning platelet biogenesis by influencing the timing of MK maturation and the efficiency of proplatelet formation [147]. Integrin α4β1 enhances thrombopoietin-induced megakaryopoiesis [143].

### 5.5. Phospholipase D (PLD)

PLD regulates actin cytoskeleton-dependent antimicrobial responses of myeloid leukocytes, including oxidant generation and phagocytosis [92]. Although platelet production was not altered, PLD1-deficient MKs displayed abnormal actin rearrangement and highly altered ultrastructures in vivo, with almost no formation of podosomes upon spreading on collagen I in vitro [148]. PLD activity is essential for podosome formation in MKs, macrophages and dendritic cells [148,149]. Studies suggest that α-actinin-1 may interact with PLD1 in fibroblasts [92,150]. After activation by RhoA and Rac1, PLD1 binds to α-actinin-1, thus facilitating the interaction between α-actinin-1 and integrin αLβ2 [151]. In cardiac muscle, PLD2 directly interacts with and is inhibited by α-actinin, a regulatory mechanism that is reversibly overcome by the small GTP-binding protein ADP-ribosylation factor (ARF) [93]. After MK/erythroid progenitors further develop into more mature MKs, phospholipase D signaling is highly expressed [152]. However, the role of phospholipase D in the process of thrombopoiesis remains to be studied.

### 5.6. Mps One Binder Kinase Activator-like 1 (MOB1)

MOB1 is an important protein in the Hippo pathway [153]. The classical Hippo signaling pathway mainly consists of large tumor kuppressor kinase (LATS)1/2 kinase, mammalian ste20-like kinase (MST)1/2 kinase, the scaffold proteins SAV1 and MOB1 and downstream transcription coactivators (Yes-associated protein (YAP) and transcriptional coactivator with PDZ-binding motif (TAZ)) [153]. The Hippo pathway plays an essential role in regulating MK proliferation, differentiation and platelet biogenesis [154,155]. In hepatocellular carcinoma (HCC) tissues, Chen reported that α-actinin-1 competitively interacts with MOB1 and decreases the phosphorylation of LATS1/YAP to regulate Hippo signaling activity [94]. Lorthongpanich et al. reported that a reduction in the activity of the Hippo core kinase LATS1/2 inhibits platelet production [155]. MOB1 is known to affect tubulin stability [156] and is significantly associated with platelet count [157]. However, whether α-actinin-1 interacts with MOB1 in MKs and platelets is an important but unanswered question.

### 5.7. LATS6 Guanine–Nucleotide Exchange Factor, Cytohesin-2 (CYTH2)

Cytohesin-2 (CYTH2, also known as ARF nucleotide-binding site opener (ARNO)), an Arf6 guanine-nucleotide exchange factor (GEF), is necessary for the development of granule neurons in the hippocampus [158]. The cytohesin inhibitor SecinH3 significantly enhances dense platelet granule secretion and aggregation [159]. Interestingly, studies conducted by van den Bosch MT et al. demonstrated that cytohesin-2 is a candidate conventional protein kinase C (PKC) substrate in platelets. Following platelet activation, cytohesin-2 is phosphorylated by PKC, leading to the inactivation of Arf6 and facilitating granule secretion [159]. Cytohesin-2 contains amino acids 386–400 (RKK RIS VKK KQE QP) at the C-terminus. α-Actinin-1 interacts with proteins with highly positively charged residues (such as RKK IK and RRF EKE KLK SQ) [160]. Using coimmunoprecipitation, Torii et al. reported that cytohesin-2 interacts with α-actinin-1 and regulates neurite extension in N1E-115 cells [95]. However, it is unclear whether the interactions of α-actinin-1 with cytohesin-2 also occur in MKs and platelets.

### 5.8. The Exchange Factor for Arf6 (EFA6)

The EFA6 family consists of four isoforms (EFA6A, EFA6B, EFA6C and EFA6D). EFA6 comprises an N-terminal disordered domain, a conserved catalytic Sec7 domain that bears nucleotide exchange activity, a pleckstrin homology (PH) domain responsible for plasma membrane localization and is involved in actin filament-based cytoskeleton rearrangement, and a C-terminal coiled–coil domain for protein–protein interactions [161]. EFA6 has been reported to be upregulated during cortical neuron maturation [162], and the functions of EFA6 are associated with neurologic disorders and human gliomas [163]. Using a two-hybrid screen, Milanini et al. reported that EFA6A could bind to the spectrin domain of α-actinin-1 through its C-terminal domain [96,164]. During lumen formation, EFA6A recruits α-actinin 1 to cortical F-actin structures through direct binding. However, there are currently no studies reporting whether the interaction between EFA6 (or EFA6A) and α-actinin-1 can affect thrombopoiesis.

### 5.9. Ataxin-2 (Sca2)

Ataxin-2 (human gene symbol *ATXN2*, protein ATXN2), a polyglutamine repeat protein, is the autosomal dominant neurodegenerative disorder disease protein of spinocerebellar ataxia type 2 (SCA2). Recent research has revealed that ataxin-2 proteins play important roles in regulating global mRNA stability and translation [165,166]. Ataxin-2 directly binds to more than 4000 transcripts, with a preference for binding to their 3′-untranslated region [167]. Genome-wide association studies revealed that single-nucleotide polymorphisms (SNPs) in the ATXN2 locus were associated with an increased risk for thrombotic antiphospholipid syndrome [168]. A recent study revealed that ataxin-2 controls the onset of terminal differentiation in megakaryopoiesis, modulates the MK transcriptome and proteome, and affects the expression of platelet surface proteins [169]. Platelets from ataxin-2 knockout mice presented increased expression of cluster of differentiation 31 (CD31) and impaired αIIbβ3 integrin-mediated platelet aggregation with phorbol myristate acetate (PMA) or with Aggretin A stimulation. In vitro glutathione S-transferase (GST)-tag pull-down experiments revealed that the CH domain at the N-terminus of α-actinin-1 binds to the N-terminus of ataxin-2 [87]. The interaction of ataxin-2 with α-actinin-1 might regulate actin-α-actinin-1 interactions since some ABD motifs of α-actinin-1 have been reported to function as signaling scaffolds and as microtubule-binding sites [170,171].

ATXN2 is highly expressed in early megakaryoblasts and is downregulated during terminal MK maturation. It associates with DEAD-box helicase 6 (DDX6) and poly(A)-binding protein (PABP), suggesting that it plays a role in the repression of mRNA translation. Depletion of ATXN2 in megakaryocytic cells (e.g., MEG-01) leads to the deregulation of mRNAs and proteins involved in platelet function and hemostasis pathways, such as integrin β3, F2R, and VAV1. In vivo, *Atxn2*^−/−^ mice presented a reduction in stage IV megakaryocytic cells and impaired platelet aggregation, specifically in response to αIIbβ3 integrin-mediated activation, despite normal platelet counts and bleeding. Thus, ATXN2 appears to fine-tune gene expression during early megakaryopoiesis, affecting downstream platelet reactivity [169,172]. However, the functional consequences of the interaction of ataxin-2 with α-actinin-1 remain to be elucidated.

### 5.10. L-Type Ca^2+^ Channel CaV1.2

The L-type Ca^2+^ channel CaV1.2 plays essential roles in cardiac contraction, gene expression and neuronal activity [173]. Binding of α-actinin-1 to the IQ motif of CaV1.2 supports CaV1.2 surface localization and postsynaptic targeting in neurons. Turner et al. reported that CaV1.2 anchoring at defined surface regions is mediated by α-actinin-1 and simultaneously increases the probability of CaV1.2 opening [97]. The EF3/EF4 domain of α-actinin-1 binds to the CaV1.2 IQ motif [97]. The EF hands of α-actinin-1 are unable to bind to Ca^2+^ under physiological conditions [174]. Therefore, the EF hands of α-actinin-1 binding to CaV1.2 are in the Ca^2+^-free state. In addition, α-actinin-2 binds to cardiac Kv1.5 channels and regulates current density and channel localization in HEK cells [175]. The primary function of CaV1.2 is well known in excitable cells, including cardiomyocytes and neurons [176]. In contrast, its role in MKs and platelets is less defined. MKs and platelets principally utilize distinct mechanisms for calcium entry [177,178,179].

### 5.11. Transient Receptor Potential (TRP) Polycystin 2 and 3

Polycystin 2 is a transient receptor potential (TRP)-type, Ca^2+^-permeable nonselective cation channel. Using a yeast two-hybrid approach, Li et al. discovered that both the N- and C-terminal domains of polycystin 2 (PC2) associate with α-actinins [98]. The PC2-α-actinin interaction leads to a substantial increase in the channel activity of polycystin-2, which is important for cell adhesion, cytoskeleton organization, proliferation and migration [98]. The transient receptor potential polycystin 3 (TRPP3)-α-actinin interaction was confirmed via yeast two-hybrid, coimmunoprecipitation and in vitro binding assays [99]. Furthermore, α-actinin not only regulates the channel function of polycystin 3 but also attaches TRPP3 to the cytoskeleton [99]. However, the direct functional roles of TRP polycystin 2 and 3 and NMDA receptors in MKs and platelets are not well established.

### 5.12. N-Methyl-D-aspartate (NMDA) Receptors

The NMDA receptor is a tetramer that consists of two obligatory NR1 subunits and two regulatory subunits [180]. The NMDA receptor is responsible for mediating Ca^2+^ influx into cells. Specifically, after Ca^2+^ enters the cell, it binds to the Ca^2+^-binding protein calmodulin to form a Ca^2+^/CaM complex. This complex then interacts with the NMDA receptor, and through this interaction, it further activates Ca^2+^/calmodulin-dependent kinase II (CaMKII) [88]. Residues 845--863 in the membrane-proximal C0 region of the NR1 subunit interact with several postsynaptic proteins, including α-actinin, CaMKII and CaM. Recent studies have shown that CaM is sufficient to displace α-actinin, which in turn promotes CaMKII binding under Ca^2+^-saturated conditions. However, CaM does not compete with α-actinin for NR1 C0 binding under Ca^2+^-free conditions [88]. NMDA receptors have emerged as pivotal regulators of MK and platelet function, directly influencing the core processes of thrombopoiesis, including MK maturation, proplatelet formation, and platelet activation. In MKs, functional NMDA receptors (composed of obligate GluN1 (encoded by Grin1), NR2A, and NR2D subunits) are essential for terminal differentiation and maturation [181,182]. Antagonizing these receptors with MK-801 profoundly inhibits the maturation of CD34^+^ progenitor-derived MKs, reducing the expression of MK-specific markers (CD41, CD61, and CD42a) and disrupting the formation of α-granules and the demarcation membrane system (DMS) [182]. This regulatory role was further validated by in vivo studies using MK/platelet-specific Grin1 knockout (PF4-*Grin1*^−/−^) mice [183]. Grin1 knockout MKs presented reduced colony formation, impaired proplatelet formation, and disrupted F-actin and α-tubulin reorganization. Transcriptomic analysis further revealed that NMDA receptor signaling influences ECM remodeling by regulating the expression of ECM components [183]. Importantly, NMDA receptor expression in MKs is maintained even in c-Mpl knockout mice (deficient in thrombopoietin (TPO) signaling), indicating that the NMDAR-dependent regulation of MK function operates independently of the canonical TPO/c-Mpl pathway [182].

### 5.13. Metabotropic Glutamate Receptor Type 5b (mGlu5b) Receptor

α-Actinin-1 was identified as a binding partner for the C-terminal domain of the mGlu5b receptor via a yeast two-hybrid screen [100]. The interaction of α-actinin-1 with the mGlu5b receptor regulates mGlu5b receptor expression on the cell surface. In addition, the interaction between α-actinin-1 and the mGlu5b receptor affects the receptor-mediated activation of the mitogen-activated protein kinase (MAPK) pathway [100]. However, whether the mGlu5b receptor is expressed and its functional role in MKs and platelets have not yet been determined.

### 5.14. Alström Syndrome Protein 1 (ALMS1)

The Alström syndrome protein ALMS1 was reported to interact with α-actinin, a component of the endosome recycling pathway [84]. The spectrin repeats and EF hands of α-actinin are important for its interaction with ALMS1 [84]. The interaction of α-actinin with ALMS1 is involved in the TGF-β signaling pathway [184]. However, whether ALMS1 is expressed in MKs and, if so, its role and interaction with α-actinin remain to be investigated.

### 5.15. Mitogen-Activated Protein Kinase Kinase Kinase 1 (MEKK1)

Direct interaction between mitogen-activated protein kinase (MAPK)/ERK kinase 1 (MEKK1) and α-actinin has been shown by residues 221--559 of MEKK1 binding to purified α-actinin in vitro [101]. MEKK1 and a-actinin form a complex in vitro and coimmunoprecipitate from 293T cell lysates [101]. Endogenous MEKK1 colocalizes with α-actinin along actin stress fibers and at focal adhesions in Madin–Darby canine kidney (MDCK) cells [101]. However, the specific role of MEKK1 in MKs and platelets has not been extensively studied.

### 5.16. Oroxylin A (OA)

Oroxylin A is a flavonoid extracted from the traditional Chinese medicine *Scutellaria baicalensis* Georgi. Oroxylin A has been reported to have antithrombotic, anti-inflammatory, proapoptotic and antitumor activities [102,185]. Oroxylin A can specifically bind to α-actinin-1 [102]. These interactions significantly prevent the activation of cancer-associated fibroblasts and restrain breast cancer metastasis [102].

## 6. Future Directions

Recent studies have revealed that mutations in the *ACTN1* gene are associated with inherited thrombocytopenia, but little is known about the exact role of α-actinin-1 in thrombocytopoiesis and its underlying mechanism. Many gaps remain to be filled in research on the mechanism of platelet production by interactions between α-actinin-1 and its partners. For example, mutations in α-actinin-1 affect the expression level of its partners during MK maturation and platelet production. Mutations in α-actinin-1 can cause thrombocytopenia, but whether mutations in α-actinin-1 affect its protein–protein interactions remains unknown. Homozygous *ACTN1*^−/−^ mice are not viable [77]. MK/platelet lineage-specific α-actinin-1 mutant knock-in should be used to investigate MK maturation and thrombocytopoiesis. To investigate the molecular mechanism underlying the role of α-actinin-1 in thrombocytopenia, the expression and localization of α-actinin-1, actin, integrin αIIbβ3 and other partners should also be examined in MKs. We hope to further elucidate the underlying mechanism of α-actinin-1 in thrombocytopoiesis and integrin αIIbβ3 signaling.

## Figures and Tables

**Table 1:** Binding partners of α-actinin-1 with known roles in megakaryocytes and platelets.

Binding Partners	Experiments	Domain of α-Actinin-1	References
Integrin β1	Affinity chromatography, solid-phase binding, peptide-pin, and pull-down assays	Rod domain	[81,82]
Integrin β3	Pull-down assays, immunoprecipitation	Spectrin-like repeats 1–2	[82]
Integrin α5	Coimmunoprecipitation	NA	[83]
Actin	Immunoprecipitation,	Actin-binding Domain	[81,82,84]
Zyxin	Blot overlay assays, solid phase binding assays	N-terminal domain	[85]
CLP36	Coimmunoprecipitation, pull-down, and yeast two-hybrid	Spectrin-like repeats 2–3	[86]
Ataxin-2	GST-tag pull-down	Calponin homology domain	[87]
NMDA receptors	GST pull-down	Calmodulin domain	[88]

**Table 2:** Binding partners of α-actinin-1 with unknown roles in megakaryocytes and platelets.

Binding Partners	Experiments	Domain of α-Actinin-1	References
Integrin β2	Solid phase binding assays, affinity chromatography experiments, and immunoprecipitation	Rod domain	[89,90]
Vinculin	Magnetic tweezers	Rod domain	[81,91]
PLD	PLD2 overlay assay, in vitro binding assay	N-terminal domain	[92,93]
MOB1	Coimmunoprecipitation, immunofluorescence	NA	[94]
CYTH2	Coimmunoprecipitation	EFh1 and EFh2 domains	[95]
EFA6	Two-hybrid screen, GST pull-down	Spectrin-like repeats	[96]
CaV1.2	NMR spectroscopy, fluorescence polarization assays, and cell surface biotinylation assays	EF3/4 domain	[97]
PC-2 and 3	Yeast two-hybrid, coimmunoprecipitation, and in vitro binding assays	Spectrin-like repeats	[98,99]
mGlu5b receptor	Yeast two-hybrid, GST pull-down, and immunoprecipitation	816–892 aa	[100]
ALMS1	Yeast two-hybrid	Spectrin-like repeats, EF hand	[84]
MEKK1	Yeast two-hybrid, immunoprecipitation	371–892 aa	[101]
Oroxylin A	Cellular thermal shift assay, drug affinity responsive target stability, and molecular docking	Calponin homology domain	[102]

## Data Availability

No new data were created or analyzed in this study. Data sharing is not applicable to this article.

## Abbreviations

The following abbreviations are used in this manuscript:

ABD	Actin-binding domain
ABSs	Actin-binding sites
ADP	Adenosine diphosphate
ALMS1	Alström syndrome protein 1
ARF	ADP-ribosylation factor
ARNO	ARF nucleotide-binding site opener
BM	Bone marrow
CaM	Calmodulin-like
CaMD	Calmodulin-like domain
CaMKII	Ca^2+^/calmodulin-dependent kinase II
CD	Cluster of differentiation
CH	Calponin homology
CMTP	Congenital macrothrombocytopenia
CHO	Chinese hamster ovary
CYTH2	Cytohesin-2
DDX6	DEAD-box helicase 6
DMS	Demarcation membrane system
ECM	Extracellular matrix
EF	E-helix and F-helix
EFA6	Exchange factor for Arf6
FGF-4	Fibroblast growth factor-4
FN	Fibronectin
GEF	Guanine-nucleotide exchange factor
GP	Glycoprotein
HCC	Hepatocellular carcinoma
HSCs	Hematopoietic stem cells
IBS2	Integrin-binding site 2
ILK	Integrin-linked kinase
ITAM	Immunoreceptor tyrosine-based activation motif
MAPK	Mitogen-activated protein kinase
MDCK	Madin–Darby canine kidney
MEKK1	Mitogen-activated protein kinase kinase kinase 1
mGlu5b	Metabotropic glutamate receptor type 5b
MK	Megakaryocyte
MOB1	Mps one binder kinase activator-like 1
MPV	Mean platelet volume
MST	Mammalian ste20-like kinase
NMDA	*N*-methyl-D-aspartate
LATS	Large tumor suppressor kinase
OA	Oroxylin A
PABP	Poly(A)-binding protein
PAR1-AP	Protease-activated receptor 1-activating peptide
PC2	Polycystin 2
PF4	Platelet factor 4
PH	Pleckstrin homology
PKN	Protein kinase N
PKC	Protein kinase C
PLD	Phospholipase D
PMA	Phorbol myristate acetate
PMF	Primary myelofibrosis
p-TEFb	Positive transcription elongation factor b
SCA2	Spinocerebellar ataxia type 2
SDF-1α	Stromal cell-derived factor 1 α
SFKs	Src family kinases
shRNA	Short hairpin RNA
SLR	Spectrin-like repeat
SNPs	Single-nucleotide polymorphisms
SR	Spectrin repeat
Syk	Spleen tyrosine kinase
TPO	Thrombopoietin
TRP	Transient receptor potential
TRPP3	Transient receptor potential polycystin 3
TXA2	Thromboxane A2
VPS33B	Vacuolar protein sorting-associated protein 33B
YAP	Yes-associated protein
ZASP	Z-band alternatingly structured protein
TAZ	Transcriptional coactivator with PDZ-binding motif
